# Hyperglycemia First Detected in Pregnancy in South Africa: Facts, Gaps, and Opportunities

**DOI:** 10.3389/fcdhc.2022.895743

**Published:** 2022-05-24

**Authors:** Ankia Coetzee, David R. Hall, Magda Conradie

**Affiliations:** ^1^Department of Medicine, Division of Endocrinology Stellenbosch University and Tygerberg Hospital, Cape Town, South Africa; ^2^Department of Obstetrics and Gynecology, Stellenbosch University and Tygerberg Hospital, Cape Town, South Africa

**Keywords:** hyperglycemia first detected in pregnancy, gestational diabetes mellitus, diabetes mellitus, antenatal predictors, postpartum care, hyperglycemia in pregnancy

## Abstract

This review contextualizes hyperglycemia in pregnancy from a South-African perspective. It aims to create awareness of the importance of hyperglycemia in pregnancy in low-middle-income countries. We address unanswered questions to guide future research on sub-Saharan African women with hyperglycemia first detected in pregnancy (HFDP). South African women of childbearing age have the highest prevalence of obesity in sub-Saharan Africa. They are predisposed to Type 2 diabetes (T2DM), the leading cause of death in South African women. T2DM remains undiagnosed in many African countries, with two-thirds of people living with diabetes unaware. With the South African health policy’s increased focus on improving antenatal care, women often gain access to screening for non-communicable diseases for the first time in pregnancy. While screening practices and diagnostic criteria for gestational diabetes mellitus (GDM) differ amongst geographical areas in South Africa (SA), hyperglycemia of varying degrees is often first detected in pregnancy. This is often erroneously ascribed to GDM, irrespective of the degree of hyperglycemia and not overt diabetes. T2DM and GDM convey a graded increased risk for the mother and fetus during and after pregnancy, with cardiometabolic risk accumulating across the lifespan. Resource limitations and high patient burden have hampered the opportunity to implement accessible preventative care in young women at increased risk of developing T2DM in the broader public health system in SA. All women with HFDP, including those with true GDM, should be followed and undergo glucose assessment postpartum. In SA, studies conducted early postpartum have noted persistent hyperglycemia in a third of women after GDM. Interpregnancy care is advantageous and may attain a favourable metabolic legacy in these young women, but the yield of return following delivery is suboptimal. We review the current best evidence regarding HFDP and contextualize the applicability in SA and other African or low-middle-income countries. The review identifies gaps and shares pragmatic solutions regarding clinical factors that may improve awareness, identification, diagnosis, and management of women with HFDP.

## Introduction

Type 2 diabetes is an intractable global epidemic and a significant societal burden, with prevalence figures escalating in parallel to obesity and ageing (1, 2). Obesity disproportionally affects women and predisposes them to type 2 diabetes (T2DM) (3) (3). South African women of childbearing age have the highest prevalence of obesity in sub-Saharan Africa, with documented prevalence figures for obesity ranging between 15.9-67.8% (4–6).

Evidence to support the concept that hyperglycemia in pregnancy affects maternal and child health beyond the ante- and perinatal period continues to emerge, with the concern that fetal exposure to hyperglycemia immortalizes the metabesity cycle (7–9). The prevalence of diabetes in the US adult population increased by 33% in the last decade. The increase in diabetes prevalence in individuals aged 30-39 years in the same period was more than double that (70%) and is evidenced by an increased prevalence of pre-existing diabetes and gestational diabetes mellitus (GDM) amongst reproductive-aged (20-39 years) women (10–12). GDM is characterized by hyperglycemia first arising in late pregnancy. It is ascribed to placental hormones that oppose insulin action and occur when the hormones reach sufficient levels to increase maternal insulin resistance (IR) from 24 weeks gestation onwards. Hyperglycemia in GDM occurs when an adequate increase in insulin secretion does not accompany the IR (13). Despite the upsurge in T2D and GDM, it is essential to note that other types of diabetes, e.g., Type 1 diabetes mellitus (T1D), monogenic diabetes, and diabetes due to steroids or transplantation, can first arise or overlap with pregnancy (14–16). Type 1 diabetes (T1D) is predominantly characterized by insulin deficiency and autoantibodies against the pancreatic β-cell at disease onset. Pregnancies complicated by T1D have increased by as much as 44% in the last 15 years (17, 18). The global prevalence of hyperglycemia in pregnancy (HIP), including both mothers with type 2 diabetes (T2DM) and GDM, was 16.9% in 2013 (19). Despite limited access to screening and potential underreporting, most live births affected by HIP occur in low- and middle-income countries (91.6%) (20).

Increased awareness and implementation of current and evolving best evidence into clinical practice will improve HIP detection, classification, and management. Subsequent enhanced maternal metabolic health is expected to have positive transgenerational legacy effects on future metabolic health and may help to curb the current pandemic of obesity and T2DM (7–9). This review aims to update hyperglycemia first diagnosed in pregnancy (HFDP) and contextualize existing knowledge from a South-African perspective.

## Pathophysiology of Gestational Diabetes Mellitus

### Normal and Abnormal Glucose Metabolism in Pregnancy

Plasma glucose concentration is the product of insulin secretion and action at any given time. Pregnancy introduces many dynamic physiological variables that alter glucose homeostasis compared to the non-pregnant state. Fasting glucose levels fall in the first trimester and are maintained in the second. Although basal plasma glucose trends are downward with further pregnancy progression, glucose peaks after meals are higher and longer (21, 22). The first trimester is characterized by increased insulin secretion and improved insulin sensitivity under elevated maternal estrogen, resulting in concomitant reductions in glucose concentration. An increased insulin effect in early pregnancy ensures optimal maternal glucose uptake and storage to supply increased energy demands later in pregnancy (22). In contrast to the early pregnancy changes in glucose homeostasis, insulin resistance (IR) increases by 30-70% in the second and third trimesters. The resistance to insulin action is mediated by several placental hormones, especially human placental lactogen (HPL), cortisol, estrogen, and progesterone (23, 24). As a result, maternal glucose uptake in muscle and adipose tissue decreases, and glucose is preferentially shunted across the placenta to optimize fetal growth. Maternal energy requirements are met by endogenous glucose production (gluconeogenesis, glycogenolysis) and *via* the breakdown of adipose tissue (lipolysis and fatty acid oxidation). Therefore, pregnant mothers are especially prone to develop hyperglycemia in the latter part of pregnancy due to an increase in IR to ensure adequate fetal nutrition. Gluconeogenesis contributes to hyperglycemia in GDM, as shown by the work of Catalano and co-workers (25). They documented that hepatic glucose production in pregnancy is suppressed to a lesser extent in women with GDM when compared to controls with normal glucose homeostasis (80% versus 95%). Increased lipolytic activity in adipose tissue, normally inhibited by insulin and stimulated by HPL, generates free fatty acids that contribute to the resistance of skeletal muscle to insulin action and, in addition, decrease the secretory capacity of the pancreatic β-cells (13).

In a euglycemic pregnancy, the physiological increase in IR is accompanied by a compensatory two-to threefold increase in insulin secretion in women with adequate pancreatic β-cell reserve (21). The appearance of GDM coincides with the period of IR in pregnancy. It is proposed that the major contributor (~80%) to GDM is β-cell dysfunction that is temporarily unmasked by the physiological IR of pregnancy (13). Evidence suggests significant heterogeneity exists in the relative contributions of insulin sensitivity and insulin secretory defects in GDM (26–28). GDM is characterized by a mild degree of hyperglycemia (fasting glucose 5.1- 5.6mmol/L, but <7mmol/L ± a 2hr oral 75-gram glucose tolerance value >7.8-8.5mmol/L, but <11.1mmol/L) (29–32). After delivery of the placenta in women with GDM, the temporary acquired, hormone-induced IR subsides. Maternal insulin sensitivity returns to pre-pregnancy levels within 1-5 days after delivery (33). Risk profiles for adverse pregnancy outcomes vary and are influenced by the degree of hyperglycemia and underlying pathophysiology (28, 34, 35).

### GDM Subtypes

Powe et al. aimed to characterize physiologic subtypes of GDM by estimating insulin sensitivity and secretion in 809 women at 24-30 weeks’ gestation (36). Compared to women with normal glucose homeostasis, women with GDM and predominant IR (51% of GDM cohort) had higher BMIs and fasting plasma glucose (FPG) values, significantly higher infant birth weights (z score = 0.57 (−0.01 to 1.37) vs birth weight z-score = 0.03 (−0.53 to 0.52) *P* = 0.001), and a greater risk of GDM-associated adverse outcomes (57.6 vvs28.2%, *P* = 0.003). These differences were independent of BMI. Women with predominant insulin secretion defects (30% of GDM cohort) had similar BMIs, FPG’s infant birth weights, and risk of adverse outcomes to the women with normal glucose homeostasis (34). Similarly, research by Benhalmina in the Belgian Diabetes in Pregnancy study showed that GDM women with higher IR represent a more unfavourable metabolic profile with a greater risk of immediate adverse pregnancy outcomes (preterm delivery, labour induction, Caesarean section, and neonatal hypoglycemia and ICU admission) (37).Again, the insulin-sensitive GDM women had similar pregnancy outcomes to normoglycemic women. Early (14 ± 4 weeks) postpartum diabetes and prediabetes rates were similar across the GDM subtypes in this study, albeit much lower (0 and 18%, respectively) than the prevalence of early postpartum diabetes in South Africa (37, 38). Miao and co-workers assessed glucose metabolism and physiological factors potentially contributing to hyperglycemia in 321 Chinese women with GDM six years after delivery. As evaluated by homeostasis model assessment (HOMA), the mechanism of hyperglycemia indicated that women with increased IR were more likely to develop prediabetes, while decreased β-cell function contributed to T2DM development (39). Ageing, elevated body mass index (BMI), and ethnicity are proposed factors contributing to IR in women with GDM (40). In a study conducted in our centre, we showed a high prevalence of overweight and obesity in women with hyperglycemia first detected in pregnancy. Increased BMI was, however, not predictive of early post-delivery T2DM (38). Ignell evaluated glucose homeostasis in a culturally diverse population after GDM and found that women of non-European origin were more insulin resistant than European women (41). The development of T2DM after GDM was higher in the non-European group, but the association between BMI and IR was discordant between Asian and Arab women (39). This implies that factors other than BMI may contribute to IR in women of different ethnic groups and socioeconomic status.

The cytokines leptin and adiponectin, secreted by adipose tissue, play an essential role in IR, T2DM, and obesity. Adiponectin enhances insulin secretion and action and therefore exhibits anti-diabetic and anti-inflammatory effects (42). Adiponectin is negatively correlated with IR and BMI. Leptin inhibits hunger and diminishes fat storage in adipocytes (43). A degree of leptin resistance occurs in T2DM and pregnancy, but the degree in GDM exceeds that of the normoglycemic pregnancy (13, 43). Amino-acid transport across the placenta also contributes to fetal macrosomia in GDM, resulting from increased placental leptin production in hyperinsulinemia and hyperglycemia (44–46).

In non-pregnant individuals, increased BMI may exist in the absence of IR, and the association between adiponectin and IR has been described as independent of a raised BMI (47, 48). In pregnancy, the human placenta is an additional source of adiponectin. Compared to normoglycemic pregnancies, lower adiponectin levels have been documented in GDM, which are associated with decreased insulin sensitivity and reduced anti-inflammatory effects (49). Insufficient placental adiponectin production influences maternal metabolism and contributes to fetal macrosomia (50, 51). In a recent South African study, pregnant women with GDM had higher BMIs, were more insulin resistant, and had lower adiponectin levels than non-GDM women (51). Interestingly, when GDM women in this study were stratified by weight, the degrees of hyperglycemia and IR were similar in the obese and non-obese individuals, with a trend towards higher adiponectin levels noted in the non-obese group. A follow-up study of the same population showed no association between the three single nucleotide polymorphisms (SNPs) within the adiponectin gene that regulates adiponectin expression (*ADIPOQ* rs266729 and rs17300539 and *MTHFR-* rs1801133). These SNPs have been associated with an increased risk for GDM in other populations (52).

### Auto-Immunity in GDM

Auto-immunity against the β-cell is traditionally linked to Type 1 diabetes mellitus (T1DM). Given the high background prevalence of obesity, many patients with a phenotype in keeping with T2DM and IR presently demonstrate autoimmunity. Prevalence figures for autoimmune diabetes (AID) in non-insulin-dependent, adult-onset diabetes range between 3.4-16% (53, 54). Autoimmunity may therefore contribute to the heterogeneity observed in HFDP and GDM. Autoimmunity is noteworthy in this high-risk, vulnerable population due to the high probability of becoming insulin dependent early in the disease course and having persistent hyperglycemia after GDM. Nillsson and colleagues indicated that 6% of women had auto-antibodies indicative of T1DM at the GDM diagnosis (55).The clinical phenotypes of the GDM antibody-positive and antibody-negative women in their cohort (n=385) were clinically indistinct at diagnosis (55). A subset of women diagnosed with GDM (± 10%, auto-antibody positive) are therefore at significantly higher risk (2-4-fold) to develop either classic Type 1 diabetes or latent autoimmune diabetes of adulthood (LADA) six months-10 years postpartum (56, 57). The prevalence of autoimmune antibodies against the β-cell, their role in diabetes development, and their association with HFDP have not been studied in South Africa. It is plausible that the IR of pregnancy unmasks auto-antibody mediated β-cell dysfunction in a subset of South-African women diagnosed with GDM.

In summary, a heterogeneity of physiologic processes underlying hyperglycemia exists among women with GDM. Increased IR appears to confer a greater risk of adverse outcomes in populations from developed countries. Most studies aimed at delineating the pathophysiological processes of GDM have been performed in West-African and Caucasian persons, with South African women grossly under-represented. The relative contribution of β-cell dysfunction, albeit on a polygenetic basis or due to autoimmunity and IR in South-African women with GDM during and after pregnancy, has not been adequately studied. A clearer understanding of these pathogenic pathways and improved knowledge regarding their relative contributions to the development of GDM may lead to more precise management strategies, including new preventative and therapeutic approaches.

## Definitions, Classification, and Screening for Hyperglycemia in Pregnancy

A variety of pathophysiological abnormalities influencing glucose homeostasis increasingly affects women of childbearing age. Historically, gestational diabetes mellitus (GDM) referred to hyperglycemia detected for the first time and during gestation that reverted to normal after delivery. The diagnostic criteria were defined primarily based on the risk of persistent postpartum maternal glucose abnormalities, with no upper threshold (58).

### Definitions and Classification

**Figure 1:**
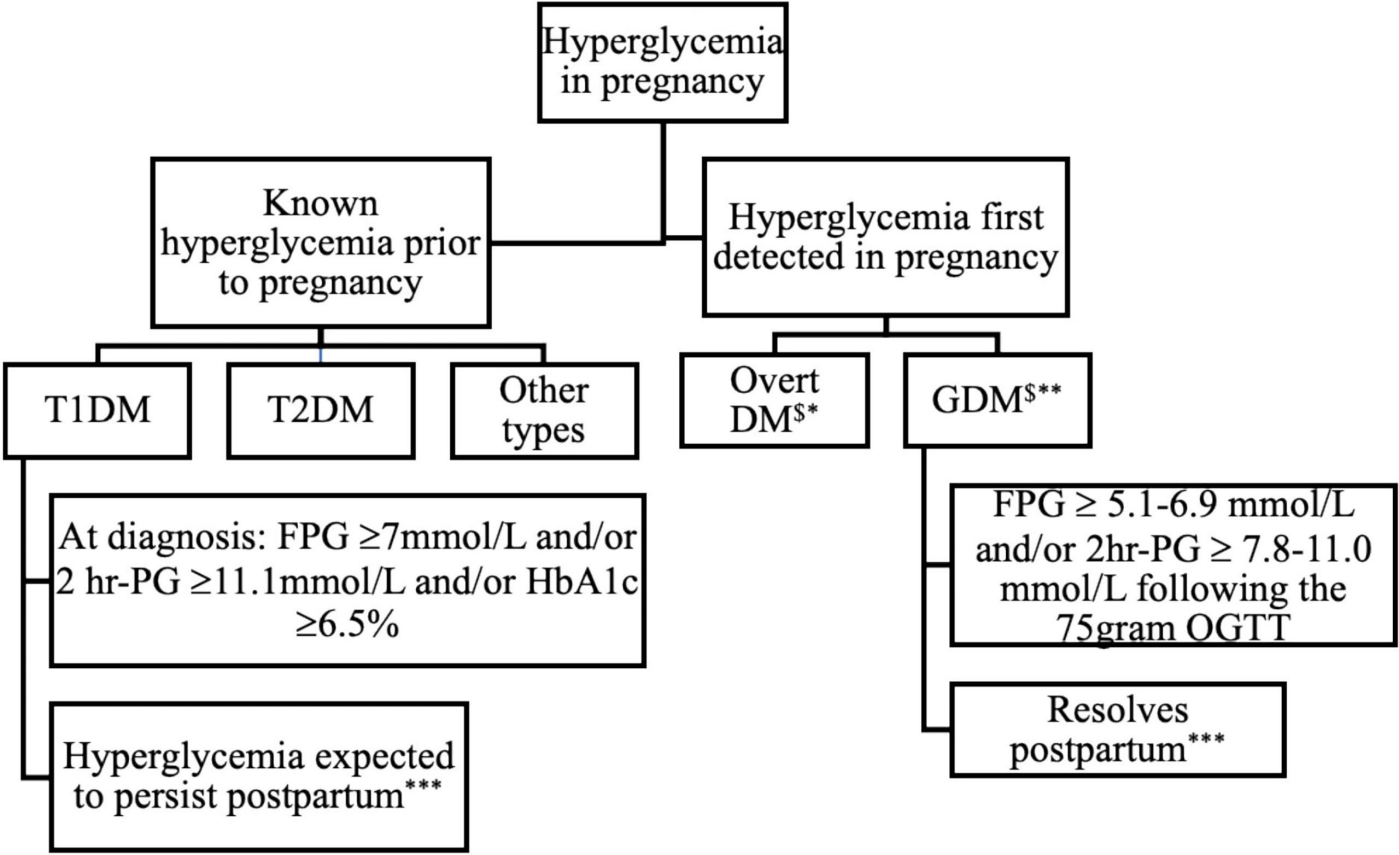
Classification and expected outcome of hyperglycemia in pregnancy. DM, Diabetes Mellitus; T1DM, Type 1 Diabetes Mellitus; T2DM, Type 2 Diabetes Mellitus; GDM, Gestational Diabetes Mellitus; FPG, Fasting plasma glucose; 2hr-PG, 2-hour plasma glucose following 75-gram OGTT, *WHO criteria **Inclusive of IADPSG and NICE criteria, *** postpartum refer to evaluation 4-12 weeks after delivery ^$^May have detectable autoantibodies.

The International Association for Diabetes in Pregnancy Study Group (IADPSG) revised the diagnostic criteria for hyperglycemia in pregnancy following the landmark Hyperglycemia and Adverse Pregnancy Outcome (HAPO) study (34, 58). Based on the degree of hyperglycemia, a gradient of risk for antenatal fetal- and maternal adversity was documented. It became increasingly apparent that gestational diabetes lacked precision regarding ante- and perinatal risk stratification (58). Recognizing these limitations, the World Health Organization (WHO) revised the terminology in 2013 (30). The phrase *hyperglycemia, first detected in pregnancy*, replaced *gestational diabetes mellitus* as the collective term for all glucose abnormalities first recognized in pregnancy (**Figure 1**). Based on the degree of hyperglycemia, they also recommended that HFDP be sub-classified into either overt diabetes in pregnancy (DIP) or gestational diabetes mellitus (GDM) (30). On the continuum of elevated glucose concentrations in pregnancy, DIP thus represents greater and GDM milder degrees of hyperglycemia associated with significantly different risk profiles, respectively.

HFDP indicates that the abnormality is first detected in pregnancy, irrespective of the degree and duration. Women with HFDP who meet the WHO criteria for diabetes in non-pregnant individuals (fasting plasma glucose (FPG) ≥7mmol/L ± 2-hour plasma glucose (2-hour PG) following a 75-gram oral glucose tolerance test (OGTT) ≥11.1mmol/L ± an HbA1c value of ≥ 6.5%) at diagnosis, are classified as having DIP (29, 30, 59). Women with HFDP who have milder hyperglycemia (FPG ≥ 5.1-6.9 mmol/L ± 2hr PG ≥ 7.8-11.0 mmol/L following the 75-gram OGTT) have GDM.

Women with overt diabetes first detected in pregnancy, and those with pre-existing known Type 1 diabetes mellitus (T1DM) or Type 2 diabetes mellitus (T2DM) are collectively categorized as DIP (30). With increased awareness regarding the subtypes of diabetes in pregnancy and recent findings of impaired insulin secretion in a subset of GDM women, it has been proposed that the most efficient way to discriminate GDM types is to include testing for pancreatic β-cell antibodies, specifically glutamic acid decarboxylase (GAD) antibody in the assessment of abnormal glucose homeostasis in pregnancy (60). Hyperglycemia in pregnancy thus includes a broad spectrum of glucose abnormalities that range from overt diabetes that predates the index pregnancy to GDM that arises during pregnancy, as depicted in **Figure 1**.

### Screening for Gestational Diabetes Mellitus

#### Universal vs Selective Screening

Globally and in South Africa, a consensus is yet to be reached regarding the most suitable screening strategy for hyperglycemia in pregnancy. Universal screening has advantages that include detecting women with milder degrees of hyperglycemia and higher overall detection rates (58, 61, 62). However, health authorities are concerned that the prevalence of GDM would be substantially increased if universal screening were to be employed and argue that milder hyperglycemia is of limited clinical significance. While this may be true regarding immediate, adverse maternal and perinatal outcomes, the associated transgenerational and future metabolic risk of milder GDM in the exposed fetus is yet to be accurately quantified (63, 64).

#### Global Screening Guidelines

Globally the screening algorithms as outlined in the National Institute of Health and Care Excellence (NICE) (2015), WHO, and IADPSG guidelines are most widely used (see **Tables 1**, **2**) (58, 68, 69). NICE and IADPSG only propose and utilize a one-step approach to screen GDM. The American College of Obstetrics and Gynecology (ACOG) accepts the former organizations’ one-step approach but recommends a two-step approach between 24 and 28 weeks as an acceptable alternative (68, 70, 71). The International Federation of Gynecology and Obstetrics (FIGO) 2015 guideline encourages universal screening for GDM using a one-step procedure and adheres to the WHO and IADPSG criteria (61, 72). The 2015 updated NICE guideline proposes the one-step screening with a 75-gram 2-hour OGTT in a select group of women (65). Primary screening at booking, irrespective of gestation, is only offered to women who had GDM in a previous pregnancy and the presence of either significant glucosuria (2+ or more) or persistent glucosuria (1+ on more than one occasion) (68). Women identified as high risk based on certain risk factors are selectively screened at 24-28 weeks of gestation (**Table 2**). The modified NICE recommendations acknowledge that GDM and T2DM are interrelated with obesity and ageing (65). Consequently, in addition to the other conventional risk factors, all pregnant women with a body mass index (BMI) ≥ 30kg/m^2^ ± older than 40 years undergo glucose testing with a 75-gram 2-hour OGTT between 24-28 weeks (68, 70).

**Table 1:** Global and local screening guidelines for hyperglycemia in pregnancy.

	NICE (2015)^#^	IADPSG	SEMDSA^$^
**At booking / gestation <24 wks**			
• Screening type	Selective	Selective	Selective
• Population to screen	Prior GDM or glucosuria^*^	High-risk women^**^	High-risk women^**^
• Methodology	75-gram 2-hour OGTT	Fasting BG or random BG or HbA1c	75-gram 2-hour OGTT
**Gestation ≥ 24 wks**			
• Screening type	Selective	Universal	Selective όr Universal (If resources allow)
• Population to screen	High-risk women^**^	All	High risk women^**^ / all
• Methodology	75-gram 2-hour OGTT	75-gram 2-hour OGTT	75-gram 2-hour OGTT

^#^Screening policy of National Institute of Care Excellence (NICE) (65)used in Western Cape, SA (31, 66). IADPSG= International Association of Diabetes in Pregnancy Study Group (29). ^$^SEMDSA, Society of Endocrinology Diabetes and Metabolism of South Africa (67). ^*^Glucosuria: refer to 2+ glucosuria on dipsticks or persistent glucosuria of 1+ on more than one occasion. ^**^High-risk women include all women with one or more risk factors, specific for the society and as noted in **Table 2**. BG, blood glucose.

**Table 2:** Risk factors for GDM used for selective screening internationally and in South Africa.

Risk factors	NICE (65)^*^	IADPSG (29)^**^	SEMDSA (67)
Glucosuria			
⚬ **Significant (2+ dipstix)**	**√**		
⚬ Repeated (1+ dipstix, > 1 visit)	√	√	√
Previous GDM	√	√	√
Family History (first-degree relative)	√	√	√
**Age > 40 years**	**√**		
Obesity (BMI > 30kg/m^2^)	√	√	√
History of polycystic ovarian syndrome	√	√	√
Women of South- Asian descent	√	√	√
High birth-weight infant ≥ 4.5kg	√	√	√
History of stillbirths of unknown cause, congenital anomalies, and polyhydramnios in present pregnancy	√	√	√
History of unexpected perinatal death	√	√	√

Risk factors prompting selective screening for GDM as defined by different guidelines. *Adopted by Western Cape Province SA; **Adopted by Gauteng Province SA. √ means "yes" or "present".

The IADPSG proposes selective screening of high-risk women at booking aimed at early detection of undiagnosed overt diabetes. Still, it encourages universal screening for GDM with a 75-gram 2-hour OGTT in pregnancy at 24-28 weeks. High-risk women, namely women with ≥ 1 risk factor as tabulated in **Table 2**, qualify for screening at first booking based on IADPSG recommendations. However, they acknowledge the need for more selective screening strategies, at booking and even at 24-28 weeks, based on locally available resources, the background prevalence of diabetes, and unique circumstances (29, 58).

The two-step approach used by the ACOG is restricted mainly to the United States, and the practice and glucose load used is unique to pregnancy. Initial screening consists of a 1-hour glucose measurement following 50g of oral glucose. A 100g 3-hour OGTT follows initial screening if the initial screening is positive (71).

#### Local Screening Guidelines

The Society of Endocrinology, Metabolism, and Diabetes of South Africa (SEMDSA), like IADPSG, encourages selective screening for GDM in high-risk women at booking (67). Unlike the IADSPG, a selective approach is also advocated for the repeat test at 24-28 weeks, i.e., to only rescreen the high-risk patients with a normal finding at booking (**Table 1**). The Society allows universal screening to be adopted in well-resourced settings, but this is seldom performed. Risk-factor-based; selective screening thus remains the predominant practice in SA at present. See **Table 2** for risk factors used for selective screening in South Africa. Even though proposed by SEMDSA, these guidelines have not been universally adopted in South Africa (67). This is attributed to well-established local practice, knowledge gaps, and the enormous patient burden with limited human and financial resources in most local public health care facilities.

The South-African healthcare landscape is heterogeneous regarding populations served and resource availability. The public sector provides health care to 84% of the population but accounts for 57% of total healthcare spending nationally, with other differences amongst provinces (73). Ideally, universal screening for GDM should occur across all health care sectors. Still, the public sector that cares for most pregnant women in South Africa has not implemented such screening due to a lack of resources (66, 74). Universal GDM screening is only achieved in well-resourced or research-based settings (74–76).

The *Gauteng Public Healthcare Sector* in South Africa abides by the recommendations of the IASDPG, and universal screening for GDM at 24-28 weeks is regarded as best practice. Two studies in the Gauteng Province’s urbanized settings documented significant but variable prevalence figures for GDM (9.1 and 25.8%) using universal screening and IADPSG criteria. Prevalence figures for GDM in the study by Adams et al. were reduced from 25.8% to 15.2% if only selective screening was done and suggested that selective screening strategies in this region might result in mild GDM cases going undetected (75, 76). Macaulay and co-workers, noted higher Caesarean section (CS) rates in GDM women. Still, there was no significant difference in birth measures amongst neonates born to GDM compared to non-GDM women in Johannesburg, South Africa (77). Most of the current guidelines for GDM balance glucose levels against the odds of adverse perinatal outcomes and highlight the need for studies that compare clinically relevant perinatal outcome parameters. This could inform local GDM practices to best guide broad policy.

Screening for GDM in the *Western Cape Public Healthcare Sector* of South Africa uses a modified version of the NICE guidelines from the United Kingdom (66, 70). The pragmatic approach supports the notion that selective screening is more cost-effective in the short term but acknowledges that long-term outcomes are neglected. Global and local screening guidelines are summarized in **Table 1**.

Screening for diabetes in the non-pregnant population of South Africa is minimal; hence, the diverse spectrum of glucose abnormalities first detected in pregnancy, including T2DM. This condition precedes pregnancy and persists after delivery. The finding that one in four South African women with HFDP has T2DM 6-12 weeks after delivery also bears witness that, in women of childbearing age, diabetes often goes undetected at the primary healthcare level until formally assessed during pregnancy (38, 78).

Adverse pregnancy outcomes associated with HFDP are proportional to abnormal glucose homeostasis’s degree, timing, and duration (78, 79). Undiagnosed pre-existing T2DM places these pregnancies in a higher overall risk category than GDM and normoglycemic women (79).To limit the time of *in-utero* exposure to hyperglycemia during the first trimester, early glucose evaluations are increasingly being recommended as part of the screening protocol for hyperglycemia in pregnancy (80).

Sesmilo evaluated first-trimester fasting plasma glucose (FPG) as a predictor of birth size and identified FPG as an independent predictor of large-for-gestation age babies, even in normoglycemic women (80). Early screening (<24 weeks) is primarily aimed at the detection of pre-existing diabetes in the clinical field, according to both the IADPSG and WHO (58, 63). Early detection of pre-existing diabetes is paramount, and immediate intervention is globally agreed upon. The management of mild hyperglycemia in early pregnancy, even though seemingly prudent, remains controversial, with no outcome-based data to support first-trimester intervention in GDM (81, 82).

## Diagnostic Tests and Criteria

### Oral Glucose Tolerance Tests and Blood Glucose Assessments

The one-step 75-gram 2-hour OGTT is the validated diagnostic test for diabetes in the non-pregnant population (59, 83). It is also accepted by most societies globally as the diagnostic test of choice for GDM, albeit with assessment at different time points and with different glycemic cut-offs (30, 58, 70). The ACOG accepts the one-step approach proposed by the former organizations but recommends a two-step approach as an alternative. [Step 1 (50-gram 1-hour) and if screen positive, Step 2 (100-gram 3-hour)] (71).

Even the one-step OGTT is a cumbersome test. Thus, practical alternatives are increasingly pursued, especially in developing countries and healthcare facilities with enormous patient burdens and limited resources, exacerbated by the recent COVID-19 pandemic. The OGTT requires the patient to be fasted overnight to obtain an FPG, then drink a standardized load of 75-gram of glucose, followed by additional blood sampling, thereby spending a minimum of 120 minutes at a health facility. In the non-pregnant state, and when screening for GDM based on NICE criteria, blood sampling is required at two-time points, i.e., at 0 and 120 minutes. A diagnosis of GDM is made if one or both blood glucose levels are in keeping with diagnostic criteria (59, 83). IADPSG guidelines require sampling at three-time points, namely at 0, 60, and 120 minutes and propose a diagnosis of GDM if any of the three predefined glycemic thresholds are met or exceeded (29).

Apart from the considerable time the patient must spend at a health facility, the OGTT also requires human resources and laboratory input. The glucose load ingested as part of the OGTT is, in addition, often associated with unpleasant symptoms such as nausea and vomiting. The need for alternative GDM testing has gained significant attention during the COVID-19 pandemic. To reduce infections, health authorities and professional bodies in the United Kingdom (UK), Canada, and Australia have issued a joint statement to limit the need for pregnant women to attend prolonged appointments, including OGTTs (84). A fasting plasma glucose (FPG) per se has been proposed as an alternative to the OGTT for screening and diagnostic purposes. Only half of the GDM cohort had impaired fasting glucose (FPG ≥5.1mmol/L) in the HAPO study. By contrast, in the other 50% of the cohort, the diagnoses of GDM were based solely on impaired glucose tolerance following the glucose load at 60 minutes ± 120 minutes (66). Recent work from our Diabetes in Pregnancy clinical research team at Tygerberg Hospital has shown that 83% of patients with more overt hyperglycemia and 56% with milder hyperglycemia are diagnosed based on fasting glucose (threshold 5.6mmol/L) (38). In a study by Nachtergaele et al., the sensitivity of 57%. (CI 0.52-0.62) when IADPSG FPG (5.1mmol/L) was compared with OGTT, improved marginally (78% (0.74-0.82)) when the FPG threshold was lowered to 4.6mmol/L (85). Dickson and co-workers compared the utility of an FPG of ≥4.5mmol/L to risk factor-based, selective screening combined with the IADPSG FPG threshold of 5.1mmol/L for diagnosing GDM in an urbanized cohort of black South-African women (85) They demonstrated that universal screening with an FPG determination and an OGTT (that was only performed if FPG ≥4.5mmol/L) had greater sensitivity and specificity (98% and 80%, respectively) in identifying GDM. Their findings proposed a universal screening strategy using FPG ≥4.5mmol/as a more efficient, cost-effective, and resource-effective method to diagnose GDM than risk factor-based selective screening in black South African women (86). Globally, it remains uncertain whether an FPG measurement alone is sensitive enough to screen and diagnose GDM.

To limit the unpleasant gastrointestinal symptoms associated with the 75-gram glucose ingestion, Marais and co-workers compared the 75-gram 2-hour OGTT with a standardized, designed breakfast (87). Overall, the 2-hour PG values of the designed breakfast correlated with the 2hr PG of the 75-gram OGTT (r=0.542, *P*<0.001), but the small study population did not allow for scrutiny at clinically relevant diagnostic glucose thresholds (87).

#### Diagnostic Criteria Based on Blood Glucose Assessments

As already explained, various diagnostic criteria for GDM exist, and consensus has yet to be reached on the optimal strategy (see the summary of criteria and classification of HFDP in **Table 3**). Most current guidelines and diagnostic cut-offs for GDM balance glucose levels against the odds for adverse fetal and perinatal outcomes and are not based solely on the risk of future T2DM (30, 58, 88). The Hyperglycemia and Adverse Perinatal Outcome (HAPO) study evaluated maternal glucose levels as less severe than T2DM in adverse perinatal outcomes. The subsequent findings shaped the more stringent diagnostic criteria for GDM currently adopted. Thus (58, 89) the widely-used IADPSG diagnostic criteria for GDM, endorsed by the American Diabetes Association (ADA) and the WHO, are based on the data obtained in the HAPO trial. The 75-gram 2-hour OGTT for GDM diagnosis by the IADPSG evaluates glucose at three-time points, namely at 0 (fasting), 60, and 120 minutes. Glucose thresholds for these time points, i.e., 5.1 mmol/L at fasting, 10 mmol/L at 60 minutes, and 8.5 mmol/L at 120 minutes, were defined based on 1.75 times estimated odds for specific, adverse perinatal outcomes (increased birth weight, increased cord C-peptide level, body fat percentage ≥ 90^th^ centile) in the multicenter, observational HAPO study (58, 89). Furthermore, the Study Group added an upper threshold to the diagnosis of GDM at the 0- and 120-minute time points to differentiate overt diabetes from GDM in women presenting with hyperglycemia for the first time in pregnancy. As glucose values are physiologically decreased in normal pregnancy, when women meet non-pregnancy diagnostic criteria (FPG ≥7mmol/L ± a 2-hour PG ≥11.1mmol/L), pre-existing diabetes is assumed (58, 59, 83). The IADPSG distinguishes between overt diabetes and GDM based on the degree of hyperglycemia at diagnosis, noting that the extent and risk of severe short-term complications are significantly greater in those with overt diabetes in pregnancy and reserves the diagnosis of GDM for the milder degrees of hyperglycemia (FPG ≥5.1- 6.9 mmol/L ± 2-hour PG ≥8.5 mmol/L-11.0 mmol/L) first arising in pregnancy.

**Table 3:** Criteria and classification of hyperglycemia first detected in pregnancy.

	WHO 2013 (30)	NICE 2015 (65)	IADPSG (29)	ACOG (71)	ADA 2015 (83)
**Method**	One step OGTT	One step OGTT	One step OGTT	Two-step OGTT	Accepts IADPSG and ACOG
**Target population**	All women	*At-risk* women	All women	All women	
**Glucose load**	75g	75g	75g	50g 1HPG screen^$^	
**Diagnostic values for diabetes in pregnancy (any 1)^#^**					
**FPG mmol/L**	≥7		≥7		≥7
**2HPG mmol/L**	≥11.1		≥11.1		≥11.1
**RPG mmol/L**	≥11.1^*^		≥11.1^*^		≥11.1^*^
**HbA1c%**	≥6.5		≥6.5		≥6.5
Diagnostic values for gestational diabetes mellitus^**^					
**FPG mmol/L**	≥5.1	≥5.6	≥5.1	≥5.3	
**1HPG mmol/L**	≥10	Not required	≥10	≥10	
**2HPG mmol/L**	≥8.5	≥7.8	≥8.5	≥8.6	
**3HPG mmol/L**	Not required	Not required	Not required	≥7.8	

^$^Followed by 100g OGTT if 1HPG ≥7.5mmol/L. WHO, World Health Organization; NICE, National Institute for Health and Care Excellence; IADPSG, International Association of Diabetes and Pregnancy Study Groups; ACOG, American College of Obstetricians and Gynecologists; ADA, American Diabetes Association; ^#^ADA requires a second confirmatory test.1HPG= 1 hour plasma glucose; 3HPG, 3-hour plasma glucose. RPG, random plasma glucose; ^*^In the presence of diabetes symptoms; ^**^1 abnormal value diagnostic except for ACOG that requires two abnormal values.

According to NICE 2015, GDM is confirmed if FPG is ≥5.6 mmol/L ± 2hPG ≥7.8 mmol/L, with no upper limit cut-off (**Table 3**) (70). The revised NICE guideline, followed by several European countries and locally at Tygerberg Hospital, Cape Town, South Africa, does not distinguish between overt diabetes and GDM (65). Therefore, the Tygerberg multidisciplinary Diabetes in Pregnancy team uses a modified version of the NICE criteria and applies the upper thresholds for diagnosing overt diabetes as in the IADPSG guideline. In a Finnish study by Koivunen et al., 4033 women were screened for GDM with a yield of 1249 (31.0%) and 529 (13.1%) with GDM according to the IADPSG and NICE criteria, respectively (90).Women with GDM based on either IADPSG or NICE criteria had a higher risk of induced delivery and cesarean section rate than controls with euglycemia. In contrast, the rate of large-for-gestational-age (LGA) babies was similar for NICE and IADPSG GDM (90). It remains to be determined whether the associated risks and pathophysiological contributors leading to hyperglycemia differ amongst women diagnosed with GDM, based on either IADPSG or NICE criteria (overt diabetes excluded). The ability of diagnostic IADPSG GDM criteria to predict adverse perinatal outcomes compared to those of NICE has not been studied in South Africa. It remains possible that the clinical relevance of the higher NICE FPG (0.5mmol/L more than IADPSG) is mitigated by, the lower 2-hour glucose threshold of 7.8 mmol/L (0.7 mmol/L lower than IADPSG) for GDM diagnosis. In the future, studies are needed to determine how these different criteria affect the perinatal outcome and future risk of diabetes in South-African women.

### Glycated Hemoglobin (HbA1c) and Albumin

Glycated hemoglobin is globally accepted as a useful modality for monitoring and evaluating longer-term control in the non-pregnant population with diabetes mellitus. In addition, an HbA1c ≥6.5% (48 mmol/mol) is regarded as one of the WHO diagnostic criteria for overt diabetes mellitus (59, 83). During pregnancy, a diagnosis of DIP is also made in women who present with HFDP if the HbA1c is ≥6.5% (48 mmol/mol) (30, 58, 59). Discrepancies as to what should be regarded as a normal HbA1c and the ideal HbA1c value for the diagnosis of diabetes remain between different populations and ethnicities (38, 89).

HbA1c in the non-pregnant population is not used to define lesser degrees of impaired glucose homeostasis and is thus not included as a diagnostic criterion for metabolic syndrome per se. Similarly, diagnostic HbA1c thresholds for GDM have not been established, and consensus has not been reached regarding the usefulness of HbA1c measurements to predict or confirm the presence of GDM. Although unsuitable for diagnosing GDM at present, HbA1c is widely used to monitor glucose control in pregnant women with GDM, Type 1, and Type 2 diabetes (30, 59, 83).

The HbA1c test has advantages, as it requires one blood sample and no fasting. All red blood cells contribute to the measured HbA1c, with levels depending on the erythrocyte lifespan, which averages 120 days in the general population. HbA1c levels decrease in pregnancy, reflecting lower maternal glucose and increased red cell turnover in pregnancy. O’Kane et al. proposed the normal reference range of HbA1c in normoglycemic pregnant women to be 4.1–5.9% (21–41 mmol/mol) (91).

Renz and colleagues evaluated the accuracy of HbA1c for GDM diagnosis and observed that while HbA1c has a high specificity, the sensitivity remained low at various thresholds (92). In a meta-analysis consisting of eight studies, the diagnostic accuracy of an HbA1c threshold of 5.7% (39 mmol/mol) had an excellent specificity (95%), which limits the usefulness as a screening test for GDM. Similarly, Nachtergaele et al. reported from France that only 26% (70/266) of women with confirmed GDM on OGTT had an HbA1c ≥5.7% (39 mmol/mol) (85). The combination of HbA1c ≥5.7% (39mmol/mol) with FPG ≥5.1mmol/L or FPG of ≥ 4.6mmol/L improves the diagnostic utility and increases sensitivity to 57% and 78%, respectively. HbA1c levels in a local GDM prevalence study noted the mean HbA1c at 24-28-week gestation to be 5.1% (5.07-5.18%) (32mmol/L; 32-33mmol/L) and 5.3% (5.21-5.39%) (34mmol/mol; 33-35mmol/L) in normoglycemic and GDM women respectively (51). During the COVID-19 pandemic, various organizations proposed HbA1c for GDM screening. The UK and Canadian interim GDM guidelines proposed an HbA1c cut-off of 5.7% (39 mmol/mol) with or without a random ± fasting glucose (84, 93). Due to limited data regarding the association between HbA1c and perinatal outcome for HbA1c values below the diagnostic threshold for overt diabetes, it is not presently regarded as a diagnostic tool for GDM (34).

The utility of HbA1c to distinguish between the various diagnostic categories (IADPSG, NICE) within the GDM spectrum remains to be elucidated. While studies from our group quantified the risk of postpartum diabetes based on antenatal HbA1c at GDM diagnosis, no study in South Africa has focused primarily on the diagnostic utility of HbA1c for GDM (94).

The measurement of HbA1c is influenced significantly by factors that influence red blood cell lifespans, such as iron deficiency or treatment with iron supplements, that affect many women during pregnancy (95). Albumin is the most abundant serum protein, and exposure to glucose leads to the non-enzymatic addition of reducing sugars to its amino acids (glycation). Albumin has a serum half-life of 2 to 3 weeks and a higher glycation rate than hemoglobin (96). Glycated albumin (GA) can thus be used as an intermediate biomarker of glycemic control in non-pregnant women and may reflect changes in the glycemic status of patients earlier than HbA1c. GA may be a practical diagnostic test and potentially provide a more accurate measure of glucose control during pregnancy (97). Caveats include the fact that serum plasma albumin decreases in normal pregnancy, reflecting hemodilution, and, being a component of the acute phase response, decreases in systemic inflammation (98, 99). Furthermore, GA measurements are affected by the presence of proteinuria, and BMI negatively influences GA levels (100). These factors must be considered when using GA as a marker of glycemic status in pregnancy. In a study by Hashimoto et al., GA levels remained stable throughout pregnancy, whereas HbA1c levels decreased during late gestation and were inversely correlated with ferritin levels and transferrin saturation (97). An accurate GA threshold for diagnosing GDM remains elusive, and the application of GA as a screening tool for GDM is therefore controversial (101, 102). The use of GA for GDM diagnosis has not been investigated in pregnant South-African women.

## Novel Biomarkers for the Diagnosis of Gestational Diabetes Mellitus

As adverse events are preventable in women with GDM, all at-risk pregnant women should have the opportunity for intervention. However, conventional screening and testing remain suboptimal and cumbersome, prompting researchers to search for biomarkers to replace the OGTT and allow universal GDM screening to become a reality (103). The biomarkers currently under investigation reflect the pathophysiological mechanisms associated with GDM (104). The prediction of GDM, GDM diagnosis, and risk stratification for the development of T2DM after GDM are areas of interest. For GDM diagnosis, metabolic (lipid, glucose, sex hormone-binding globulin, adipocytokines), inflammatory [tumour necrosis factor-alpha, Interleukin-6 (Il-6), C-reactive protein (CRP)], placenta-derived (cytokines including adipokines and inflammatory, glucose transporters), genetic and epigenetic biomarkers are all subjects of intense research and interest to the scientific and clinical GDM community (105).

### Metabolic Biomarkers

Intermediary metabolism is intricately linked to adipose tissue and mediated by adipose-derived factors or adipokines. Leptin and adiponectin have been discussed earlier (see pathophysiology section). Overall, the adipokines’ association with obesity, inflammation, T2DM, and GDM are well elucidated, but their value is limited by lack of specificity (105).

### Inflammatory Biomarkers

Markers of inflammation associated with GDM are also involved in the IR of euglycemic pregnancies. IL-6 is a promising biomarker that may improve the early prediction of GDM. Tumour necrosis factor-alpha (TNF-alpha) may indicate IR and contribute to β-cell dysfunction. The association between TNF-alpha and other surrogate markers of inflammation such as c-reactive protein is also strengthened obesity in GDM (106). Collectively, inflammatory cytokines are central to the pathogenesis of GDM, but their utility to diagnose GDM is limited by the lack of distinction between association and causality.

### Placental Factors

More knowledge on placental factors (pregnancy-associated plasma protein-A (PAPP A), placental growth factor, as well as sex-hormone-binding globulin (SHBG) and their respective roles in GDM, is required to add value to clinical practice (106, 107).

### Genetic Biomarkers

Genetic variants and single nucleotide polymorphisms (SNPs) are significantly associated with T2DM in genome-wide association studies (GWAS) and recently in GDM (108–110). Variants in four maturity-onset diabetes of the young (MODY) genes and the PDX1 gene have also been implicated in GDM development (52, 111). In a study on black South-African women, Botha and colleagues assessed 16 single nucleotide polymorphisms (SNPs) in five MODY and the PDX1 gene. They found a significant inverse association between women with the minor allele of rs4581569 in PDX1 and GDM (111). Dias and co-workers investigated genetic polymorphisms associated with adiponectin expression. They found that *ADIPOQ*rs266729 and rs17300539 and *MTHFR* rs1801133 polymorphisms are not associated with GDM in a population of black South African women. The author concludes that limited access to genetic testing, the role of gene-gene and gene-environment interactions, and the small study samples limit extrapolation of these findings to clinical practice.

Telomeres, the non-coding caps at the ends of chromosomes, are shortened by oxidative stress and inflammation and are associated with the development of diabetes (112). Many studies report telomere shortening in placentas from pregnancies with uncontrolled diabetes and GDM (113–115). Telomere shortening is also linked with future diabetes and cardiovascular disease in mothers and offspring (116–118). A better understanding of telomere homeostasis in GDM may illuminate some of the mechanisms underpinning health and disease’s developmental origins. Still, again, this has yet to be studied in South-African women with GDM (119, 120).

### Epigenetic Biomarkers

Epigenetic changes are increasingly implicated in the pathophysiology of metabolic diseases, including GDM (110). Intra-uterine exposure to famine and over-nutrition in the form of hyperglycemia results in an epigenetic change. Epigenetic mechanisms regulating and altering gene expression and function include DNA methylation, microRNA (miRNA), and histone modifications (110, 121, 122). There is tremendous plasticity during development, and in response to environmental factors, DNA methylation is the most thoroughly studied epigenetic mechanism (122). Bouchard and co-workers found that lower DNA adiponectin promoter methylation levels are correlated with higher maternal IR and glucose levels after OGTT during the second and third trimesters in GDM (122). Locally, Dias et al. investigated global DNA methylation in 63 (138 controls) women with GDM and reported the association between obesity and serum adiponectin (123). They noted no difference in DNA methylation between women with GDM and normoglycemic women. Their finding might be because global DNA methylation is too crude to detect subtle but significant differences associated with mild hyperglycemia in pregnancy (HIP) in their population, suggesting the need for gene-specific methylation (123). Subsequent gene-specific methylation analysis by the same authors indicated that a total of 1046 cytosine-phosphate-guanine sites (CpG) (associated with 939 genes) were associated with GDM. DNA methylation of the top five CpG loci showed distinct methylation patterns in GDM and correlated with glucose concentrations. One CpG site mapped to the calmodulin-binding transcription activator 1 (CAMTA1) gene, which has been shown to regulate insulin production and secretion, may offer potential as an epigenetic biomarker in the South-African population (124).

The second most studied epigenetic mechanism, Micro-RNA, has been identified as a significant regulator of β-cell mass, β-cell function, and lipid metabolism (125). Circulating microRNAs are proposed as promising diagnostic biomarkers and predictors of complications in T2DM (124–126). In the last decade, the relevance of microRNAs in GDM has been studied extensively. Zhu et al. identified a microRNA signature composed of five microRNAs (miR-16-5p, miR-17-5p, miR-19a-3p, miR-19b-3p, and miR-20a-5p) were upregulated in the plasma of women affected by GDM (126). Tagoma et al. identified 15 upregulated miRNAs involved in 41 metabolic pathways that included insulin signalling in women with GDM (127). In South-African women with GDM, the expression of two of these microRNA’s, miR-20a-5p and miR-222-3p, was decreased (128). In subsequent work, the same authors studied the expression of these microRNAs in HIV-positive pregnant women with and without GDM (GDM n=15; non GDM n=52). They found no difference in expression between the two cohorts (129).

While the genetic and epigenetic landscape may provide diagnostic biomarkers with clinical utility in GDM, both globally and in South-African women, the clinical use, at present, remains limited. Further validation of these novel biomarkers is needed to elucidate their potential role in women with GDM.

## Significance of Hyperglycemia in Pregnancy: Immediate and Long-Term Consequences and Management

It is well established that hyperglycemia in pregnancy (HIP) adversely impacts maternal and fetal health. Antenatal complications include maternal hyperglycemic emergencies, potential teratogenic and growth abnormalities, and even death of the fetus. A pregnancy complicated by hyperglycemia also indicates a significantly increased future risk for the mother to continue having or developing impaired glucose homeostasis and even cardiovascular disease later in life (130, 131). Just as concerning is the possible trans-generational effect of *in-utero* exposure of the growing fetus to hyperglycemia, with a significantly increased risk for metabolic sequelae, including T2DM in later life (132–134).

### Immediate Consequences: Antenatal and Perinatal

In the short term, ante- and adverse perinatal outcomes include congenital abnormalities, miscarriage (both early), preterm birth, macrosomia or growth restriction, stillbirth, hypertensive disorders of pregnancy, birth trauma, cesarean section, neonatal hypoglycemia, and jaundice (135). These fetal and maternal complications generally correspond to the degree of hyperglycemia, the timing and duration of conception, and organogenesis (136–138). The risks associated with GDM and adverse perinatal outcomes were quantified by the landmark Hyperglycemia and Adverse Pregnancy Outcome (HAPO) study (89). This multicentre, multi-ethnic observational study that included 23316 pregnant women found that maternal hyperglycemia, less severe than diabetes, was associated with limited but important adversity. These adverse effects occurred irrespective of maternal obesity and included hypertensive disorders in the mother, cesarean delivery, fetal adiposity, increased neonatal birth weight (above 90^th^ centile), raised cord-blood C-peptide levels, and neonatal hypoglycemia. The results of that study formed the basis of the 2010 IADPSG recommendations on the diagnosis and classification of hyperglycemia in pregnancy (58). Their revised diagnostic glucose values for GDM represented values at which infant birth weight, cord C-peptide and percent body fat > 90^th^ percentile reached 1.75 times the estimated odds of these outcomes, based on fully adjusted logistic regression models. There are limited data on neonatal outcomes after GDM in sub-Saharan Africa, but macrosomia appears to be a common complication (139). Macaulay et al. found no difference between neonatal birth measures in GDM‐exposed and unexposed neonates in Soweto, South Africa (77). Mild hyperglycemia, timely and appropriate treatment, as well as earlier delivery (GDM 38 (37–39) weeks vs non-GDM 39 (38–40) weeks (*P* = 0.001) might explain this. Interestingly, a gender-specific sub-analysis showed that GDM exposure was associated with increased fetal growth measures, especially abdominal circumference in males, evident at 16–18 weeks’ gestation (77).

Data on the perinatal outcome of patients with pregestational diabetes and GDM attending Groote Schuur Hospital, Cape Town, South Africa, indicate preterm delivery rates of 38.7% for women with T2DM and 34.9% for GDM (140). In that retrospective audit, the perinatal mortality rate was 2.5% (25/1 000 births), with pre-existing diabetes (T1D and T2DM) contributing to most deaths (6.4% and 4.2% within the subgroups, respectively). The overall rate of congenital malformations was 2.4% (n=18 cases), 1.5% for GDM (within generally accepted background incidence), and 4.6% and 5.7% for T2DM and T1D, respectively. In a retrospective audit from the neighbouring tertiary institute in the Western Cape Province, Tygerberg Hospital, lower preterm delivery rates (17.4% and 8.4% in mothers with T2DM and GDM) were recorded (141). Most women in the latter study were overweight or obese (GDM 95%; T2DM: 70%), and chronic hypertension was present in 23% and 42% with GDM and T2DM. Seven cases of fetal anomalies occurred in mothers with T2DM (7.6%), and the combined perinatal mortality rate for T2DM and GDM was 5.3% (52.6/1000 births), with only one early neonatal death (141).

During pregnancy, the variation in glucose metabolism impacts the ability to maintain good glycemic control and predisposes women to medical emergencies such as diabetic ketoacidosis (DKA), associated with fetal mortality rates as high as 27% (142, 143). A pregnant woman oscillates between the fed and fasted state, with the latter characterized by “accelerated starvation.” Alternate fuels are provided for the mother during pregnancy, while glucose is reserved for the fetus (144). Increased IR in pregnancy is interrelated with a perceived intracellular state of starvation, with resultant endogenous glucose production and lipolysis that generates free fatty acids (FFAs) (145, 146). FFAs lead to ketosis and increase maternal IR with decreased insulin secretion based on lipotoxicity through reduced β-cell function (13).

The DKA risk in pregnancy is further amplified by any condition exacerbating insulin demand. A particular danger is that DKA recognition is often delayed in women without known pre-existing diabetes. Schneider et al. noted infection (27%) and omission of insulin therapy (18%) to be the most common precipitants of DKA in 2025 pregnant women in the US (143). DKA without significant elevation in blood glucose levels, i.e., euglycemic DKA, is also more common in pregnancy, a diagnosis often missed or delayed if not considered. Euglycemic DKA represents between 0.8% and 1.1% of all DKA cases in pregnancy (143, 145, 146). The lower-than-expected glucose concentration is attributed to physiologic hemodilution, increased placental glucose transporters, glomerular filtration with glycosuria, and a decrease in glycogenolysis and hepatic glucose production.

### Long-Term Implications for Mothers and Offspring

A pregnancy complicated by hyperglycemia indicates a significantly increased risk for the mother to have persistent glucose abnormalities after delivery or to develop abnormal glucose homeostasis and even T2DM later in life (147). This risk is dependent on the degree of antenatal hyperglycemia and the duration of follow-up (148). However, even amongst women diagnosed with GDM based on IADPSG criteria, the risk of developing T2DM was increased more than three-fold after 11 years of follow-up (OR=3.4; GDM:52%; non-GDM:20%) and after adjusting for maternal variables (149). Our group demonstrated a high prevalence (26%) of T2DM 6-12 weeks after delivery in women with antenatal HFDP using NICE criteria (38). Chivese et al., in a cross-sectional study performed 5-6 years after delivery on 220 women with GDM in Cape Town, South Africa, indicated that 48% of women had T2DM. At the time of the study, the mean age of the women was 37.2 years (SD 6.0), and 47% of participants were unaware of their diabetes status (150).

The impact of GDM on the increased future risk of cardiovascular (CV) disease, irrespective of the development of T2DM, has recently been assessed in a systematic review and meta-analysis by Kramer, Campbell, and Retnakaran (151). GDM conferred a 2.3-fold increased risk of CV events in the first decade postpartum. Thus, even without developing T2DM, GDM identifies a vulnerable population and provides an opportunity for timely CV risk modification. Data on CV risk in South-African women with HFDP is limited to a single study. Nicolaou et al. documented a ten-fold increased risk to develop T2DM 3-6 years postpartum (4.6 and 27.6-fold for GDM and DIP, respectively) in black African women with HFDP (152). They noted a higher mean carotid intima-media thickness, a surrogate marker for atherosclerotic CVD in women with HFDP compared to normoglycemic pregnant women.

More extensive studies with control groups and longitudinal surveillance programs after GDM are urgently needed to define and mitigate CV risk in South-African women.

### Transgenerational Effects on Offspring

In 2017, one in seven live births globally were affected by hyperglycemia in pregnancy (HIP) (20). Several epidemiological studies have since noted detrimental effects of exposure to maternal GDM on adipose tissue distribution and documented an increased risk for T2DM in young offspring exposed to HIP (147, 153). The HAPO follow-up study evaluated glucose metabolism in 4832 ethnically diverse women at 24-28 weeks gestation (149, 154). Higher intra-uterine glucose was linearly associated with offspring glucose levels [maternal FPG with child Impaired Fasting glucose (IFG) and maternal 1-h and 2-h PG with child Impaired Glucose Tolerance (IGT)]. Eleven percent of the offspring of mothers with GDM had IGT compared with 5% of mothers without GDM. The GDM exposed adolescents were insulin resistant (adjusted mean difference -76.3%, CI -130.3 to -22.4) and had limited β-cell compensation as evidenced by an oral disposition index (−0.12, CI −0.17 to −0.064) compared with the offspring of mothers without GDM (149, 154).

In the Exploring Perinatal Outcomes among Children (EPOCH) study, GDM exposure was strongly associated with more significant IR during childhood and adolescence, even after adjustment for BMI (155). This supports the notion held by many clinicians involved with the care of reproductive-aged women that HIP is perpetuating the increasing global burden of obesity, metabolic syndrome, and T2DM. Proposed mechanisms by which *in-utero* exposure to hyperglycemia influences the risk of T2DM in the offspring point towards epigenetic modifications, changes in insulin receptor expression and function, adipokine metabolism, and β cell development (156–158). DNA methylation is an epigenetic marker that has been implicated in the observed long-lasting metabolic effects following intrauterine exposure to hyperglycemia. Shiau and co-workers published the first study investigating the association between prenatal GDM exposure and DNA methylation in 3–10-year-old offspring in the Tianjin GDM Observational study (159). They found that offspring of mothers with GDM exhibit premature epigenetic ageing by accelerated DNA methylation, which is associated with cardiometabolic risk in the GDM youth compared to controls. Locally, Soepnel and co-workers assessed the association between HFDP (GDM and DIP) and child obesity in pre-school-aged children in Johannesburg, South Africa. The prevalence of overweight/obesity was 10.5% and 11.1% in the 3–6-year-old children exposed to GDM and DIP, respectively, while only 3.9% in the offspring of normoglycemic mothers (160). Fat mass indices were significantly higher in offspring exposed to DIP but attenuated when maternal BMI was adjusted. In another South African study performed in Cape Town, Chivese evaluated the anthropometry of the offspring exposed to HFDP at birth and pre-school age. At birth, 29.6% of the neonates were classified as large for gestational age (LGA), and 12.2% had macrosomia. The prevalence of LGA and macrosomia mirrored the degree of antenatal hyperglycemia, with a higher prevalence in neonates exposed to DIP compared to GDM. The combined prevalence of overweight and obesity was 26.5% at pre-school age. Maternal third trimester 2-hour PG was associated with z-scores for birthweight and pre-school age weight, indicating that this population’s antenatal 2-hour PG was a modifiable risk factor (161).

In a longitudinal study conducted in the PIMA Indian population, maternal hyperglycemia was the strongest predictor for T2DM in youth. Maternal hyperglycemia accounted for 40% of T2DM development in offspring evaluated at age 5-19 years (162). Children exposed to GDM *in utero* are at risk of being insulin resistant with limited β-cell compensation compared with the offspring of mothers without GDM. The exposure is significantly and independently associated with childhood IGT, occurring over and above the genetic predisposition and persisting after accounting for adiposity (162). This implicates a transgenerational and potential intergenerational effect of exposure to *in-utero* hyperglycemia on offspring’s metabolic function and CV risk. It remains to be elucidated to what extent and mechanism T2DM development in the offspring is influenced by the degree of antenatal glucose exposure and the epigenetic changes arising in the placenta of the mothers with hyperglycemia.

## Postpartum Maternal Screening Following Pregnancy With Hyperglycemia

The characteristic mild hyperglycemia associated with GDM resolves within hours after delivery. Unfortunately, the maternal risk does not conclude at that time. Following GDM, the risk of developing T2DM increases up to seven-fold, dependent on the duration of follow-up, with at least a two-fold higher risk to develop CV disease, independent of T2DM. A previous pregnancy complicated by GDM also increases the risk of recurrent GDM in subsequent pregnancies (163, 164). Due to these findings and because unrecognized diabetes classified as GDM may not be apparent immediately following delivery, follow-up assessment after GDM and interpregnancy metabolic care has been identified as a fixable gap in women’s preventative healthcare. The WHO, ADA, ACOG, and NICE endorse the need for postpartum glucose evaluation unequivocally but differ regarding the best modality for screening (OGTT or FPG) and diagnostic cut-offs (See **Table 4**) (30, 31, 59, 83, 165). In the Translating Research into Action for Diabetes (TRIAD) study, FPG alone as a screening modality only identified one-quarter of participants with diabetes *following GDM (* 166*). *The postpartum WHO diagnostic criteria for prediabetes differ from the ADA criteria. The WHO defines IFG as a value of ≥ 6.1mmol/L to < 7mmol/L, whereas the ADA defines the lower threshold for diagnoses at 5.6 mmol/L (59, 83).

**Table 4:** Postpartum screening after Hyperglycemia First Detected in Pregnancy.

	WHO (30)	NICE (65)	ADA (83)	ACOG (71, 88)
**Method**	FPG or 75-gram 2-hour OGTT	FPG	OGTT	FPG or 75-gram 2-hour OGTT
**Target population**	All women	All women	All women	All women
**First postpartum evaluation**	6 weeks	6 weeks	4-12 weeks	
**Subsequent evaluations, if the first evaluation normal**	Annually		Every three years	
**Subsequent evaluations if IFG^#^ or IGT**			Annually	

IFG, Impaired fasting glucose; IGT, Impaired glucose tolerance. WHO, World Health Organization; NICE, National Institute for Health and Care Excellence; ACOG, American College of Obstetricians and Gynecologists; ADA, American Diabetes Association; ^#^ADA ≥5.6mmol/L.

The 75-gram 2-hour OGTT is presently regarded as standard practice and ensures the best sensitivity to identify patients with any degree of abnormal glucose homeostasis. Data on the prevalence of sustained dysglycemia in South African women following a pregnancy complicated by GDM is limited to a few studies. In a retrospective audit of the outcome of diabetic women in Soweto, Huddle et al. reported persistence of glucose intolerance in 8.3% of predominantly African patients that were evaluated six weeks postpartum (167). In a study from our group, of 181 women with GDM evaluated at six weeks postpartum, one in four (n=47/181, 26%) had diabetes and 15% prediabetes (n=28/181) based on IGT in more than two-thirds (n=19/28; 68%) (78). Thus, two out of five patients in this cohort had persistent abnormal glucose homeostasis early postpartum, making it likely that pre-existing T2DM was included in the GDM diagnosis. In a prior study, also from Cape Town, our group showed an overall 46% (n=36/78) prevalence of abnormal glucose homeostasis after GDM (diabetes in n=21/78; 27%) and prediabetes in 19% (n=15/78) (38). Retrospectively, 37% (n=29/78) of GDM women had evidence of overt diabetes in pregnancy as determined by the current gold standard IADPSG criteria (78). In a multivariate analysis of this cohort, a family history of diabetes, HbA1c at diagnosis, and age were robust antenatal predictors of postpartum diabetes (38). Following the first wave of the COVID-19 pandemic, increased attention is being paid to implementing strategies to minimize patients’ time at healthcare institutions. The HbA1c as an alternative to postpartum OGTT has been recognized as an easy, cost-effective, and less time-consuming postpartum glucose assessment, even before COVID-19. Kim et al. compared HbA1c ≥ 5.7% with FPG and 2hr PG after OGTT in women with recent GDM (6 weeks-36 months) and found the agreement levels acceptable for detecting abnormal glucose homeostasis (168). In another study, a similar HbA1c, i.e., between 5.7-6.4% to diagnose prediabetes after GDM, detected an additional 10.6% of patients compared to OGTT (169). Claesson et al. found the optimal cut-off HbA1c value for predicting abnormal glucose tolerance to be 5.2% (33mmol/mol) with a sensitivity and specificity compared with an OGTT of 69.2% and 59.7%, respectively (170). In the Atlantic Diabetes in Pregnancy (Atlantic DIP) partnership, women diagnosed with GDM by IADPSG criteria were assessed postpartum. A combination of FPG 5.6mmol/L and an HbA1c ≥5.7% (39mmol/L) yielded a sensitivity of 90% and specificity of 84% for detecting abnormal glucose homeostasis 1-year post GDM with the OGTT as the comparator (171). Katreddy et al. documented the significant risk for early postpartum diabetes with an HbA1c ≥6% (≥42 mmol/mol) (172). Weinert et al. also demonstrated an elevated risk in the presence of an HbA1c ≥ 6% (42 mmol/mol) in Brazil (173). Considering the ethnic disparities in HbA1c, even in the non-pregnant setting, HbA1c must be compared with an OGTT in South-African women following GDM to determine the optimal cut-off for diagnosis (174, 175).

In a local cohort of women with GDM, our group noted that the increased risk for early postpartum diabetes witan an HbA1c ≥ 6.2% (44mmol/mol) at diagnosis and in the month before delivery was four-fold and five-fold (94). Women with fasting and a 2-hour PG above IASDPG GDM cut-off values emerged as another high-risk category for postpartum diabetes (78).

The hypothesis that T2DM is preventable by lifestyle intervention in high-risk patients is supported by extensive observational studies that underpin the importance of postpartum glucose testing. In the landmark Diabetes Prevention Program in the US (DPP), T2DM in an ethnically diverse population with prediabetes, was reduced by 58% with intense lifestyle change versus a 31% reduction obtained with the anti-diabetic agent metformin. When the persistence of these effects was investigated ten years after DP, the risk for developing diabetes was reduced by 34% in the lifestyle group and 18% in the metformin group compared with placebo in non-pregnant patients (176). Interestingly, women with prior GDM had a more significant reduction in the development of diabetes (55%) with lifestyle only. Metformin was also more effective in the GDM cohort, with a 50% risk reduction compared to14% in the non-GDM matched group (177). The identification and intervention strategies in patients with prior GDM constitute a unique window of opportunity for early detection, intervention, and reduction of the future burden of T2DM. Globally, however, fewer than half of women with GDM undergo postpartum OGTT testing (178). Locally, low follow-up rates after GDM also prevail, with only 46% of women attending the OGTT 4-12 weeks after delivery (78). The high prevalence of postpartum diabetes (27%) and low postpartum retention at our institution underscores the importance of identifying the women with the highest risk. This will facilitate the optimal use of available diabetes prevention and early management resources. To overcome the problem of low postpartum follow-up rates after GDM and improve efficiency in the healthcare system, Wessels et al. investigated in-hospital post-delivery glucose and its ability to replace the 4–12-week OGTT (179). In contrast to similar studies, early post-delivery glucose was not an alternative to OGTT testing 4-12 weeks after delivery. This was partly due to a significant improvement in maternal fasting glucose in all sub-categories of glycemia 24-72hrs after delivery.

## Conclusion

In South Africa, antenatal evaluation and management of GDM vary amongst healthcare sectors and levels of care. Following a diagnosis of HFDP, postpartum OGTT testing is mainly limited to tertiary and research settings and sub-optimally attended. Antenatal factors that best predict postpartum diabetes are the degree of hyperglycemia at GDM diagnosis, abnormal glucose values on OGTT, and a family history of diabetes. The prevalence of diabetes 4-12 weeks after delivery ranges from 9-25.8%, depending on the antenatal GDM screening practice and diagnostic criteria. At the same time, the incidence of diabetes increases with the duration of postpartum follow-up. It is alarming that 46% of women with HFDP have diabetes 5-6 years after delivery, with many unaware of their diagnosis.

The mechanisms involved in GDM, DKA, and T2DM are heterogeneous. They include variances in β-cell function (insulin secretion) ± insulin action ), as evidenced by studies on genetic, epigenetic, and other biomarkers in South-African women. Further studies of South African women with HFDP and their offspring are needed to improve our understanding, assessment, and management of this potentially preventable gateway to diabetes. Current research gaps to be addressed include DKA risk, the contribution of autoimmune diabetes, ideal postpartum assessment, and future diabetes and CV risk. These aims are in line with global sustainable development goals and may influence the management of HFDP through health policy aimed at optimal detection, prevention, and control.

## Author Contributions

All three authors have met the requirements for authorship. AC and MC summarized the manuscript. All authors contributed to the article and approved the submitted version.

## Conflict of Interest

The authors declare that the research was conducted without any commercial or financial relationships construed as a potential conflict of interest.

## Publisher’s Note

All claims expressed in this article are solely those of the authors and do not necessarily represent those of their affiliated organizations, or those of the publisher, the editors and the reviewers. Any product that may be evaluated in this article, or claim that may be made by its manufacturer, is not guaranteed or endorsed by the publisher.
